# Supplementary material for: Ultrahigh dynamic range and low noise figure programmable integrated microwave photonic filter
Source: Nat Commun. 2022 Dec 17;13:7798. doi: 10.1038/s41467-022-35485-x (PMC9759590; doi:10.1038/s41467-022-35485-x)
Supplement: Supplementary file 1 — Supplementary Information [file 41467_2022_35485_MOESM1_ESM.pdf]

Supplementary Information for Ultrahigh Dynamic Range and Low Noise Figure Programmable Integrated Microwave Photonic Filter

Okky Daulay,[1,*] Gaojian Liu,[1,2,*] Kaixuan Ye,[1] Roel Botter,[1] Yvan Klaver,[1] Qinggui Tan,[2] Hongxi Yu,[2] Marcel Hoekman,[3] Edwin Klein,[3] Chris Roeloffzen,[3] Yang Liu,[4] and David Marpaung[1,†]

[1] *Nonlinear Nanophotonics Group, MESA+ Institute of Nanotechnology University of Twente, Enschede, Netherlands*
[2] *China Academy of Space Technology (Xi'an), Xi'an, China*
[3] *LioniX International BV, Enschede, Netherlands*
[4] *Institute of Physics, Swiss Federal Institute of Technology Lausanne (EPFL), CH-1015 Lausanne, Switzerland*

SUPPLEMENTARY INFORMATION A: MODULATION TRANSFORMER

A modulation transformer (MT) is an optical device designed to independently shape and synthesize arbitrary optical modulation spectrum. Such device constructed from multiple optical elements, namely spectral de-interleaver built from an asymmetric Mach-Zehnder Interferometer (aMZI) with three ring resonators topology [1], a tunable attenuator, a phase shifter and a combiner (Supplementary Fig. 1). This device takes any conventional phase or intensity modulation spectrum as an input and synthesize an output optical modulation spectrum with components (optical carrier and sidebands) entirely independent in phases and amplitudes [2]. This technique is different to prior techniques which mainly focus on PM-IM or IM-PM transformation by tailoring the phase of optical carrier.

The two output ports of spectral de-interleaver consist of one isolated sideband in one channel and an optical carrier with the remaining sideband in the other channel. Then, the isolated sideband is routed to cascaded tunable attenuator and phase shifter to independently tailor its phase and amplitude. Last, we re-combine this tailored isolated sideband with the rest of the optical spectrum component (optical carrier and unprocessed sideband) to synthesize different modulation spectrum with designer phase and amplitude relations between its spectrum elements.

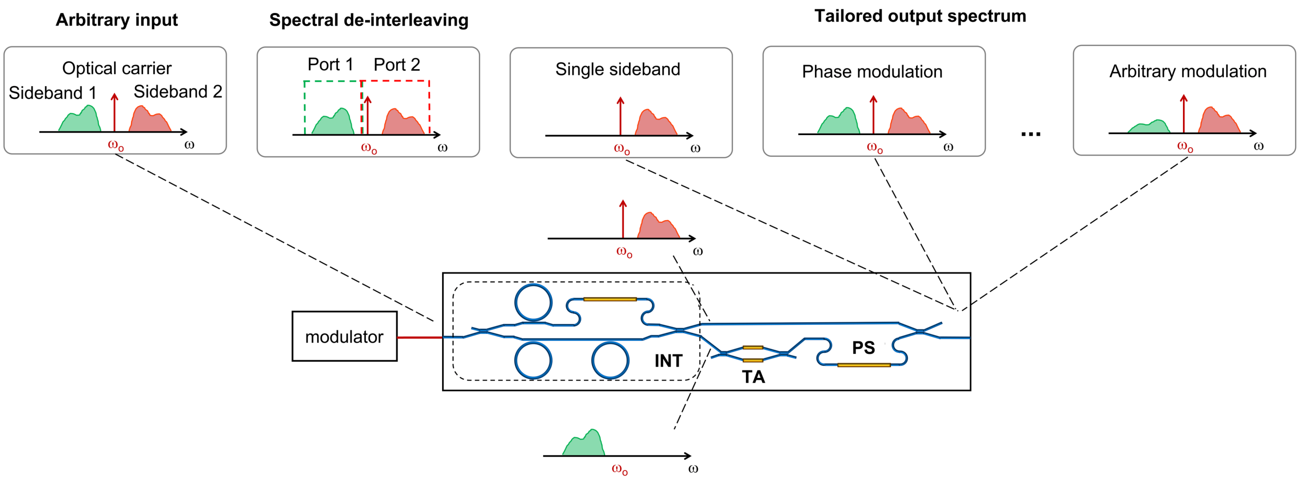

Supplementary Fig. 1. Operational principle of Modulation Transformer. Schematic and operational principle of the modulation transformer (MT). The spectral de-interleaver is used to isolate one sideband from the optical carrier and the other sideband. A cascaded of tunable attenuator and a phase shifter is used to tailor the phase and amplitude of the isolated sideband. A combination with the optical carrier and unprocessed sideband leads to versatile spectral shaping, synthesizing variety of modulation format, such as single-sideband (SSB) modulation, phase modulation, and arbitrary modulation. INT: Spectral de-interleaver, TA: tunable attenuator, PS: phase shifter.

* These authors contributed equally

† david.marpaung@utwente.nl

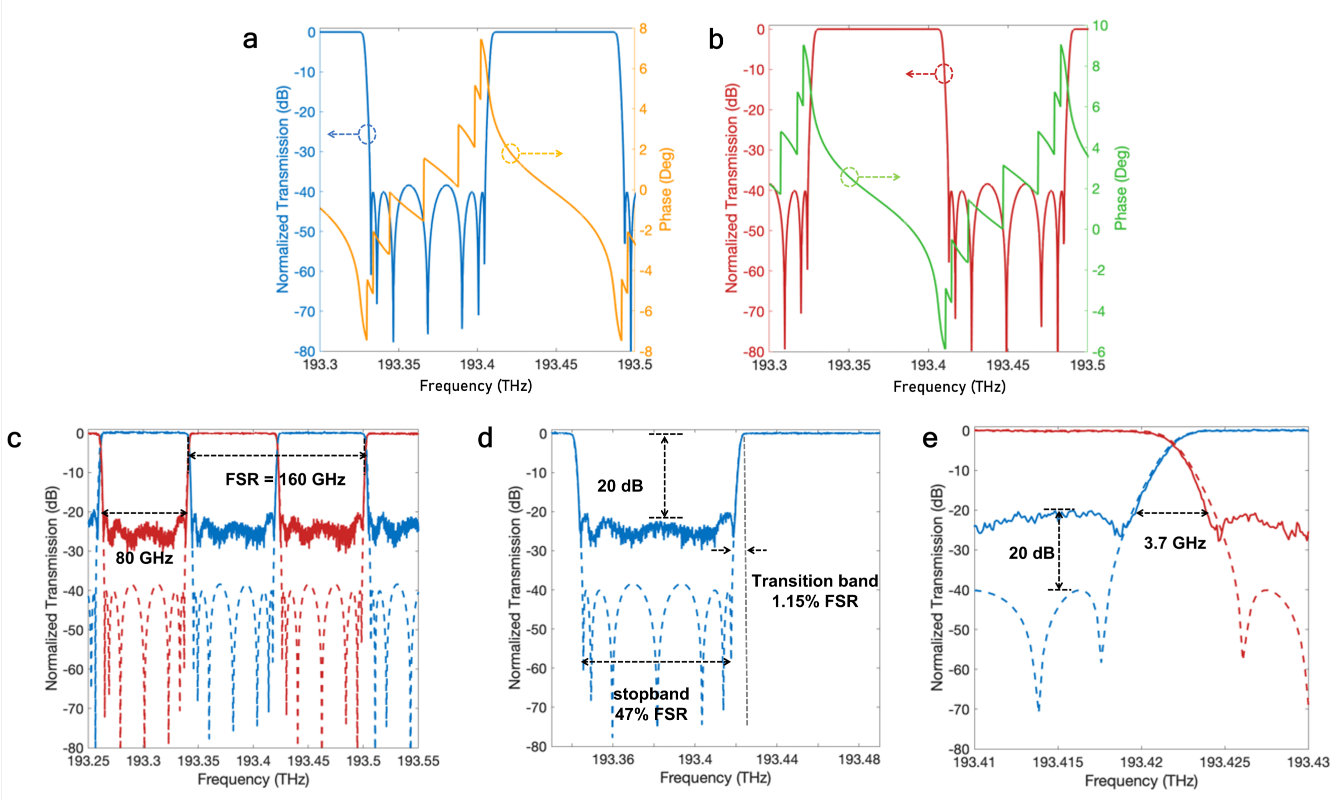

Supplementary Fig. 2. **Spectral de-interleaver responses.** **(a)** Simulation of amplitude and phase of the bar port from a spectral de-interleaver constructed using an asymmetric Mach-Zehnder Interferometer (aMZI) and three ring resonators. **(b)** Simulation of amplitude and phase from the cross port of spectral de-interleaver. **(c)** Fitting of simulation (dashed lines) and experimental (solid lines) results of spectral de-interleaver. **(d)** The rejection of spectral de-interleaver is 20 dB with stopband 47% and transition band 1.15% of spectral de-interleaver's FSR. **(e)** The transition between bar and cross port of spectral de-interleaver is 3.7 GHz.

The mathematical description of each optical building block in the MT can be described as follows, First, a phase shifter is written as

$$H_{ps}\left(z\right) = e^{-j\phi} \tag{1}$$

then, a tunable coupler used for a tunable attenuator can be described as

$$H_{tc}\left(z\right) = \begin{bmatrix} \sqrt{0.5} & -j\sqrt{0.5} \\ -j\sqrt{0.5} & \sqrt{0.5} \end{bmatrix} \begin{bmatrix} 1 \\ e^{-j\phi} \end{bmatrix} \begin{bmatrix} \sqrt{0.5} & -j\sqrt{0.5} \\ -j\sqrt{0.5} & \sqrt{0.5} \end{bmatrix} \tag{2}$$

last, a spectral de-interleaver built from three ring resonators assisted aMZI structure can be expressed as [3]

$$H_{bar} = A_{11}A_{12}H_{11}\left(z\right) - A_{13}A_{14}H_{22}\left(z\right) \tag{3}$$

$$H_{cross} = A_{21}A_{22}H_{11}\left(z\right) - A_{23}A_{24}H_{22}\left(z\right) \tag{4}$$

where,

$$
\begin{aligned}
H_{11}(z) &= T_1(\omega)\, T_3(\omega) \\
H_{22}(z) &= T_2(\omega)\, H_{\Delta L}(z)\, H_{ps}(z) \\
H_{\Delta L}(z) &= \gamma z^{-1} \\
A_{11} &= c_3 c_4 e^{-j\phi_2} - s_3 s_4 \\
A_{12} &= c_1 c_2 e^{-j\phi_1} - s_1 s_2 \\
A_{13} &= c_4 s_3 e^{-j\phi_2} - c_3 s_4 \\
A_{14} &= c_1 s_2 e^{-j\phi_1} - c_2 s_1 \\
A_{21} &= c_3 c_4 e^{-j\phi_2} - s_3 s_4 \\
A_{22} &= c_2 s_1 e^{-j\phi_1} - s_1 s_2 \\
A_{23} &= c_3 c_4 - s_3 s_4 e^{-j\phi_2} \\
A_{24} &= c_1 s_2 e^{-j\phi_1} - c_2 s_1
\end{aligned}
$$

Here, c and s are the cross-coupling coefficient and the self-coupling coefficient of tunable element in the spectral de-interleaver respectively. Then, the amplitude response ($T(\omega)$) of the ring resonator used in the spectral de-interleaver can be expressed as [4]

$$T(\omega)\, e^{-j\theta} = \frac{a - c_r e^{-j\psi(\omega)}}{1 - a c_r e^{-j\psi(\omega)}} e^{-j(\pi + \psi(\omega))} \tag{5}$$

where $c_r = \sqrt{1-k}$, $a = 10^{-\alpha L/20}$, k, α, L, and ψ are the self-coupling coefficient, the single-pass amplitude of the optical ring resonator, the coupling coefficient, the propagation loss of optical waveguide (dB/cm), the round trip length of ring, and the round-trip phase respectively. Similarly, we can define $s_r = \sqrt{k}$ as the cross-coupling coefficient.

Supplementary Fig. 2(a) and 2(b) show the simulated phase and amplitude responses from bar and cross port of spectral de-interleaver respectively. It is critical to synthesize a "box-shaped" response, flat-top to precisely isolate optical modulation spectrum elements (optical carrier and sidebands). The spectral de-interleaver has a linear phase response in the passband, while the phase in the stopband shows a nonlinear response, which may cause signal distortion in the MT. Supplementary Fig. 2(c) shows 160 GHz of spectral de-interleaver's free spectral range (FSR) with 80 GHz of bandwidth and rejection of up to 20 dB. The transition band in this response is 1.15% of spectral de-interleaver's FSR or 1.85 GHz as shown in Supplementary Fig. 2(d) with 3.7 GHz of transition bandwidth between bar and cross port as shown in Supplementary Fig. 2(e). These results show the narrowest transition band reported in a spectral de-interlever device [5].

While the stopband rejection within this spectral range is measured to be 20 dB (Supplementary Fig. 2(d)), this measured rejection is roughly 20 dB higher than the simulated rejection of 40 dB in Supplementary Fig. 2(e) as it is assumed in the simulation that the power coupling coefficients and the loss of all the couplers are in the optimized values. However, it is difficult to achieve identical stopband rejection in a physical measurement, because typically, a fabrication process add more losses in the waveguide and unoptimized couplers, leads to the limitation in the stopband rejection.

Next, the bar port of spectral de-interleaver is connected to a cascaded tunable attenuator and phase shifter, where the cross port of spectral de-interleaver is sent to a waveguide directly to a combiner. Last, a combiner at the output of the MT is used to re-combine the output of cascaded tunable attenuator and phase shifter with the output of the waveguide from the cross port of spectral de-interleaver to synthesize different modulation spectrum.

In this work, two examples are given to show modulation transformation process, such as intensity-to-phase modulation (IM-PM) transformation and phase-to-intensity modulation (PM-IM) transformation. Such modulation transformation can be mathematically described as [3]

$$\begin{aligned} E_{low}(t) &= H_{tc} \begin{bmatrix} 0 \\ H_{ps} H_{bar} E_{in}(t) \end{bmatrix} \\ &= 0.5 \begin{bmatrix} e^{-j\pi} - 1 & -j\left(e^{-j\pi} + 1\right) \\ -j\left(e^{-j\pi} + 1\right) & 1 - e^{-j\pi} \end{bmatrix} \begin{bmatrix} 0 \\ H_{ps} H_{bar} E_{in}(t) \end{bmatrix} \\ &= H_{ps} H_{bar} E_{in}(t) \\ &= e^{-j\pi} H_{bar} E_{in}(t) \\ E_{up}(t) &= H_{cross} H_{wg\Delta L} E_{in}(t) \\ E_{comb}(t) &= E_{low}(t) + e^{-j\pi/2} E_{up}(t) \end{aligned}$$

where $E_{low}(t)$, $E_{up}(t)$, $H_{wg\Delta L}$, H_{bar}, H_{cross} and $E_{comb}(t)$ are the output field of lower channel after spectral de-interleaver, upper channel after spectral de-interleaver, transfer function of waveguide at upper channel of the MT, transfer function of spectral de-interleaver's bar port, transfer function of spectral de-interleaver's cross port and the synthesized spectrum after signal combination respectively, and $e^{-j\pi/2}$ states a $\pi/2$ phase shift induced by the cross transfer function of the tunable coupler.

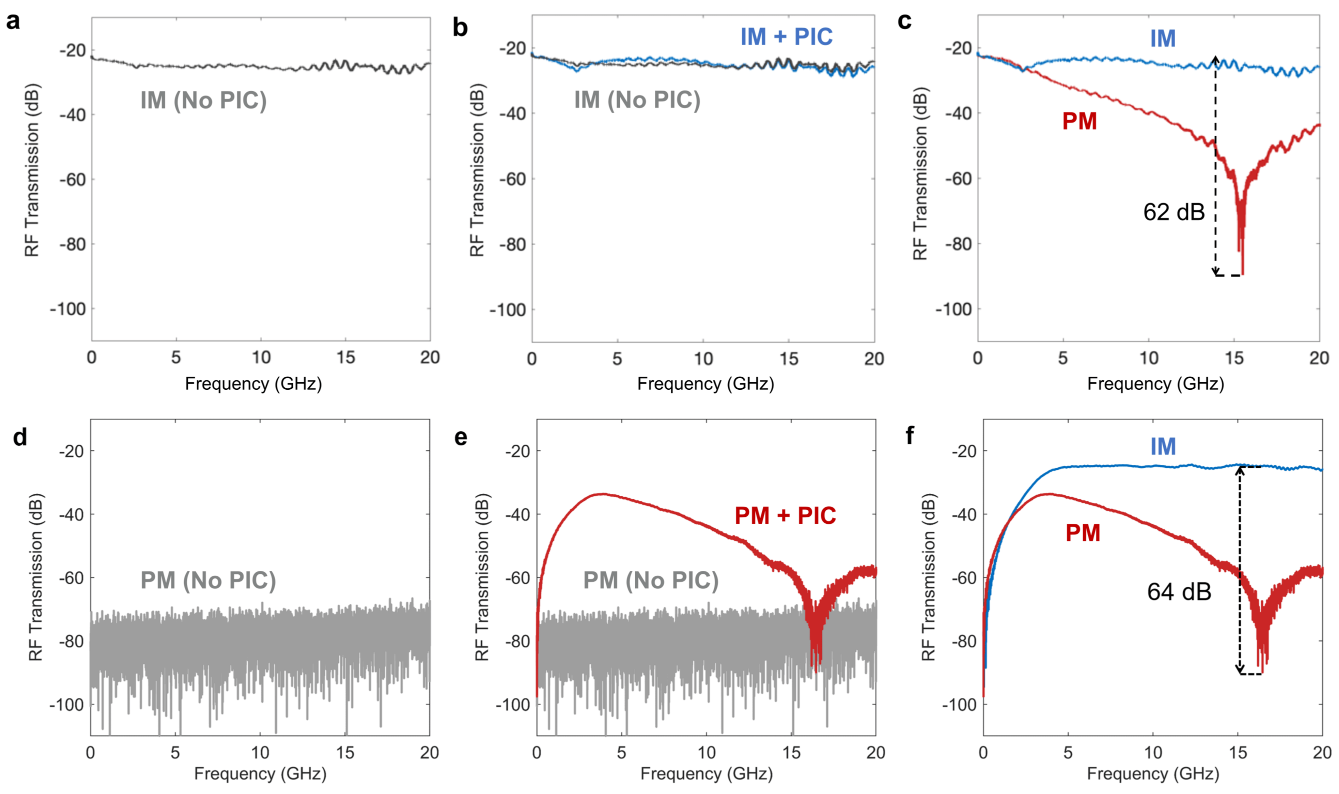

Supplementary Fig. 3. **Experimental results of intensity-to-phase modulation (IM-PM) and phase-to-intensity modulation (PM-IM) conversions.** **(a)** RF magnitude response of the intensity modulation (IM) link without the photonic integrated circuit (PIC) as a benchmark. **(b)** RF magnitude response with the PIC (solid blue line). Here, the PIC response is set to be similar with the benchmark. **(c)** Conversion from IM (blue) to phase modulation (PM) (red) with optimized extinction of 62 dB. **(d)** RF magnitude response of the PM-based photonic link without the PIC as benchmark. **(e)** RF magnitude response of the PM link with the PIC (solid red line). **(f)** Conversion from PM (red) to IM (blue) with optimized extinction of 64 dB.

.

We controlled the MT in our circuit to synthesis two different modulation schemes using a PM and an IM spectrum as an input . In the first experiment, we sent an IM spectrum directly to a photodetector in a simple photonic link setup (photonic link: a laser source, a Mach-Zehnder modulator (MZM) and a photodetector), and measured the RF transmission for a reference. Then, we process the IM spectrum using the MT in our circuit (photonic link: a laser source, a MZM, an erbium-doped fiber amplifier (EDFA), a photonic integrated circuit (PIC) and a photodetector), and convert into PM spectrum. Supplementary Fig. 3(a) shows the IM spectrum in the RF domain as the reference signal in a simple photonic link setup. Next, the IM spectrum is sent to our circuit containing of a MT as shown in Supplementary Fig. 3(b). Here, we try to match the RF spectrum (with PIC) with the reference spectrum (without PIC). While both of the sidebands in IM spectrum are in-phase, the measured RF transmission is high (solid blue line in Supplementary Fig. 3(b) and 3(c)). Then, we tailored the phase of isolated sideband by tuning the phase shifter in the MT. Last, the processed isolated sideband is re-combined with the optical carrier and remaining sideband to synthesized the PM spectrum (solid red line in Supplementary Fig. 3(c)) with 62 dB extinction.

In second experiment, we used the MT for PM-IM transformation with the same principle as prior experiment, but with the PM spectrum as an input. Supplementary Fig. 3(d) shows the transmission of PM spectrum as a benchmark in a simple photonic link setup with direct detection (photonic link: a laser source, a phase modulator (PM) and a photodetector). Then, we sent the PM spectra into our circuit (photonic link: a laser source, a PM, an EDFA, a PIC and a photodetector), where the RF spectrum is shown in Supplementary Fig. 3(e). When both sidebands are out of phase, the measured RF transmission is low (solid red line in Supplementary Fig. 3(e) and 3(f)). Then, we tuned the phase shifter in the MT to rotate the phase of isolated sideband. Eventually, an IM spectrum is synthesized after the signal recombination, and it is corresponding to a maximum RF transmission (solid blue line in Supplementary Fig. 3(f)) with 64 dB extinction. In these two experiments, the operational bandwidth and extinction are mainly limited by the roll-off and the dispersion of the spectral de-interleaver, notably at the transition band (operational frequency of < 5 GHz).

SUPPLEMENTARY INFORMATION B: DOUBLE INJECTION RING RESONATOR

The use of an optical circuit named double-injection ring resonator (DI-RR) is aimed to provide various unique responses in a ring resonator-based optical circuit from single output. The underlying idea of this circuit is to inject two, mutually coherent, optical signals of the same wavelength into a single add-drop ring resonator from single input [6] as illustrated in Supplementary Fig. 4(a). To optimize such circuit for our device, we further investigate the variety of unique phase and amplitude response synthesized by the DI-RR, which is a key parameter for programmable integrated MWP circuit.

Mathematically, the model describing the transmitted electric field dependence on the wavelength of the DI-RR is given by

$$E_{t1}(\lambda) = \frac{\left(\tau_1 + \tau_2^* \alpha e^{-j\theta}\right)}{1 - \tau_1 \tau_2^* \alpha e^{-j\theta}} \left|E_{j1}(\lambda)\right| e^{-j\phi_{j1}} - \frac{K_1 K_2^* \sqrt{\alpha} e^{-j\theta}}{1 - \tau_1 \tau_2^* \alpha e^{-j\theta}} \left|E_{j2}(\lambda)\right| e^{-j\phi_{j2}} \tag{6}$$

where $\tau = \left|\tau\right| e^{-j\psi_\tau}$ is the transmission of the directional coupler, $K = \left|K\right| e^{-j\psi_K}$ is the coupling coefficients of the directional couplers, α is the loss coefficient of the ring, E_j is the injected fields, and ϕ_j is the injected fields phases. θ is the phase accumulated by the light traversing the ring at steady state that described as

$$\theta(\lambda) = \frac{2\pi}{\lambda} n_{eff}(\lambda) L_{ring} \tag{7}$$

with λ being the wavelength, L_{ring} the length perimeter of the ring, and n_{eff} the effective index of the propagating mode. Supplementary Fig. 4(b) - 4(g) depict the measured RF phase and amplitude responses of the six different functions synthesized from the DI-RR with free spectral range (FSR) of 20 GHz, such as notch filter (Supplementary Fig. 4(b)), bandpass filter (Supplementary Fig. 4(c)), triangular response (Supplementary Fig. 4(d)), sawtooth response (Supplementary Fig. 4(e)), all-pass response (Supplementary Fig. 4(f)) and Fano-like response (Supplementary Fig. 4(g)). The inset of each figure depict the response obtained from high resolution optical spectrum analysis. The characterization is important to have a full understanding of the DI-RR's potential in the system.

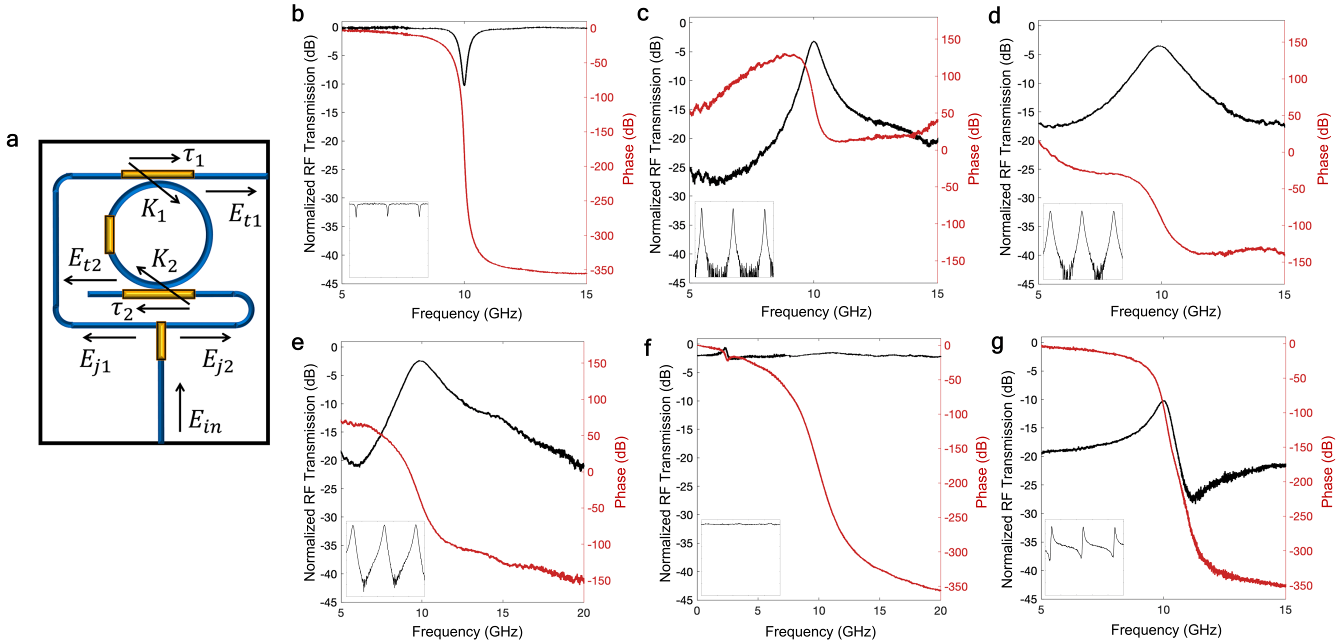

Supplementary Fig. 4. RF phase and magnitude responses of the DI-RR. (a) Schematic of double injection ring resonator. **(b)** Notch filter. **(c)** Bandpass filter. **(d)** Triangular response **(e)** Sawtooth response. **(f)** All-pass response. **(g)** Fano-like response. The inset of each figure shows the corresponding optical response.

SUPPLEMENTARY INFORMATION C: EXTENDED EXPERIMENTS

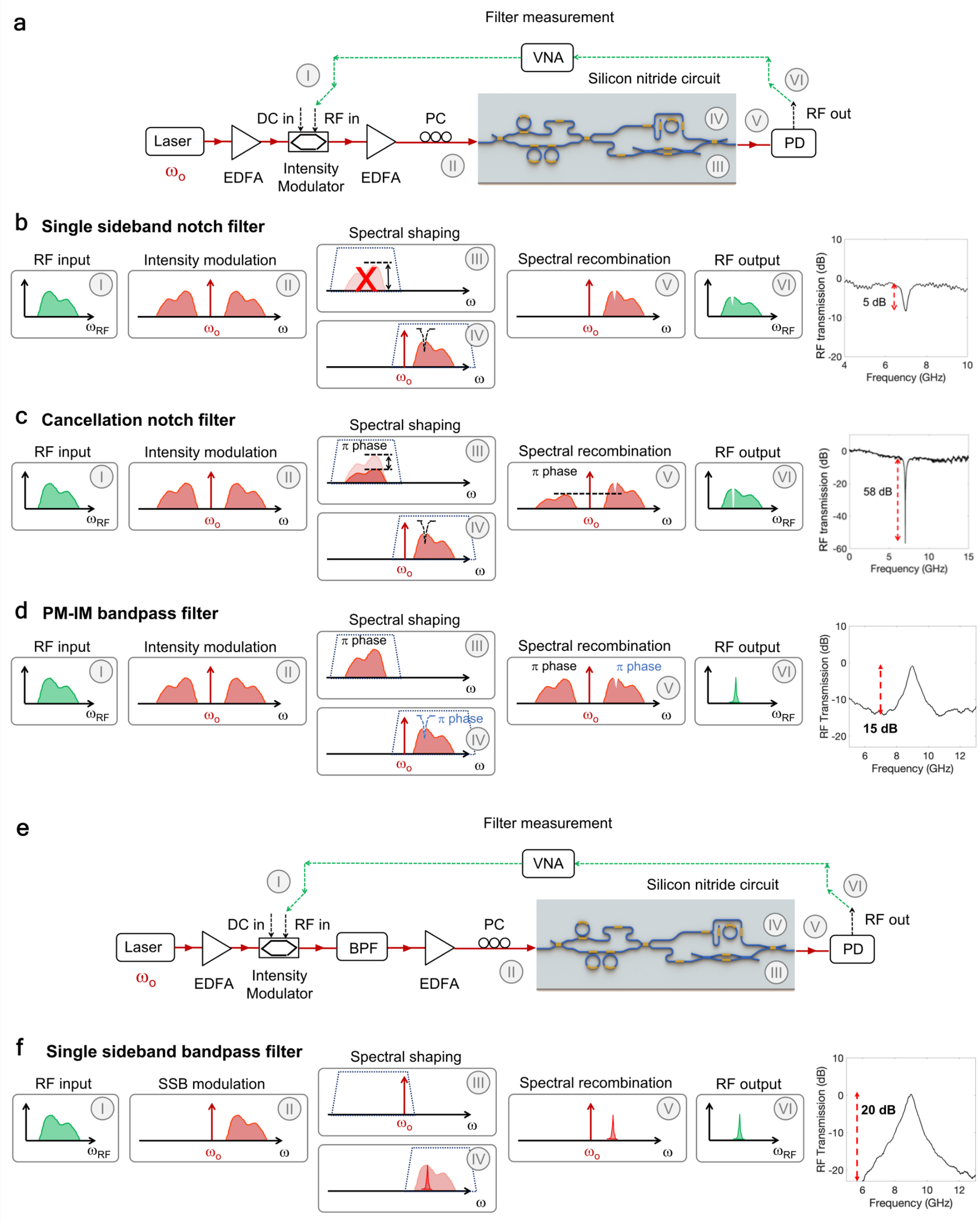

Supplementary Fig. 5. **Extended experiment scenarios.** **(a)** Experiment setup for three different filtering scenarios. The modulation transformer (MT) and the double-injection ring resonator (DI-RR) are programmed to exhibit different functions using a common intensity modulation (IM) input. **(b)** Scenario 1: Single sideband (SSB) notch filter. The MT is used to synthesize SSB modulation and the DI-RR to synthesize 5 dB-deep notch response. **(c)** Scenario 2: Cancellation notch filter. MT is used to create asymmetric dual-sideband modulation conversion while DI-RR shows 5 dB-deep notch response. Destructive interference at the notch frequency amplifies the RF notch filter response to 58 dB. **(d)** Scenario 3: PM-IM bandpass filter (BPF). The MT is used to create phase modulation (PM). The over-coupled (OC) notch response from DI-RR breaks the PM condition and creates a BPF response. **(e)** Experiment setup used for demonstrating the SSB BPF. **(f)** Scenario 4: SSB bandpass filter. The MT is used to re-insert an optical carrier to mix with a SSB response that has been filtered using the DI-RR.

To demonstrate the feasibility of the MT together with the versatility of DI-RR in programmable integrated MWP circuit, we conducted experiments with an intensity modulation (IM) spectrum as the input optical spectrum. Four different scenarios were applied to demonstrated the versatility of the circuit. Supplementary Fig. 5(a) shows the experiment setup and Supplementary Fig. 5(b) - 5(e) show the operation principle and application of four different scenarios of the proposed device. In the system, the input RF signal is upconverted to optical domain with intensity modulator generating single frequency optical carrier and two identical amplitude sidebands that are in-phase.

In the first scenario, we convert an intensity-to-single sideband (SSB) modulation to, later on, create a SSB RF notch filter as shown in Supplementary Fig. 5(b). An IM signal is sent to pass through a spectral de-interleaver in the MT, which spatially isolates one sideband in the spectrum from another sideband and optical carrier. Then, the isolated sideband was sent to tunable attenuator to be fully attenuated, where the other sideband and optical carrier were sent to a DI-RR which synthesized a notch response. Last, the output of DI-RR was combined with the fully attenuated sideband and exhibited a SSB RF notch filter.

Then, in the second scenario, an IM signal is sent to the MT, perform intensity-to-asymmetric dual sideband (aDSB) modulation conversion and synthesize high rejection RF notch filter. Here, the attenuation of isolated sideband is similar with the rejection of notch response synthesized by the DI-RR. Then, the phase shifter was used to create phase difference (0 - π) between two sidebands relative to optical carrier. In parallel, the other sideband and optical carrier were processed by the same notch response as in the first scenario. It is important that the rejection of notch response from the ring to be equal with the amplitude's difference between two sidebands. The idea is to have a desctructive interference between aDSB together with notch response from DI-RR to amplify the rejection of synthesized RF notch filter as shown in Supplementary Fig. 5(c).

Next, in the third scenario, we conducted phase-to-intensity modulation (PM-IM) conversion technique to create RF bandpass filter. Here, we converted the modulation spectrum from intensity-to-phase modulation (IM-PM). During the process, we only changed the phase of the isolated sideband by controlling the interconnected phase shifter from 0 - π. While, the other sideband and optical carrier were processed using notch response of DI-RR set at over-coupling (OC) regime. After IM-PM conversion using MT, due to OC state in the DI-RR, a π-phase shift is introduced at the desired notch frequency, creating constructive interference for phase-to-intensity (PM-IM) modulation conversion and synthesized RF bandpass filter as shown in Supplementary Fig. 5(d). Previously, this approach can only be done in a phase modulator-based MWP system.

Last, another RF bandpass filter is exhibited using optical carrier re-insertion technique with the MT. In this approach, a SSB modulation is synthesized using the external bandpass filter (BPF) to cancel out one sideband in IM spectrum using experiment setup depicted in Supplementary Fig. 5(e). Then, the SSB modulation was sent to the spectral de-interleaver in the MT and split into two outputs containing only optical carrier in one output and one sideband in the other. Then, the sideband was sent to a DI-RR which tuned and synthesized a bandpass response. The RF bandpass filter is constructed while the optical carrier is re-injected to the processed sideband using a combiner at the output of MT as shown in Supplementary Fig. 5(f).

SUPPLEMENTARY INFORMATION D: MAXIMUM RF GAIN OF RF NOTCH FILTER

The maximum RF gain of the RF notch filter stays above 0 dB over the entire frequency range of 5-20 GHz. Supplementary Table 1 and Supplementary Fig. 6 show the RF gain at various frequencies.

Supplementary Table 1. The maximum RF Gain of the RF notch filter over the entire frequency range

**Freq. (GHz)**	**RF Gain (dB)**	**Freq. (GHz)**	**RF Gain (dB)**	**Freq. (GHz)**	**RF Gain (dB)**	**Freq. GHz**	**RF Gain (dB)**
5	4.23	9	2.60	13	2.71	17	1.61
6	4.01	10	2.39	14	2.05	18	1.19
7	3.62	11	2.16	15	2.60	19	1.14
8	2.87	12	2.72	16	2.11	20	1.71

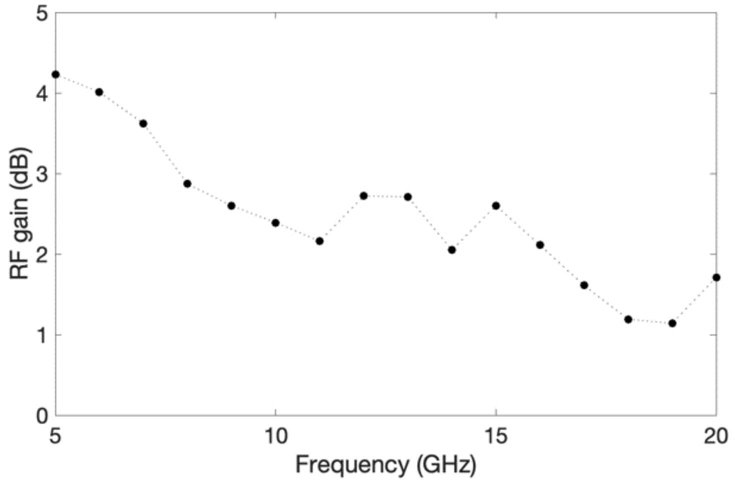

Supplementary Fig. 6. **The RF gain of the RF notch filter.** Plot of the maximum RF gain of the RF notch filter over the entire frequency range.

SUPPLEMENTARY INFORMATION E: THEORETICAL ANALYSIS OF LINEARIZATION METHOD

A theoretical analysis is performed to find the requirements for linearity improvement in the proposed RF notch filter. We first analyze the relation between optical spectrum and the beating products after photodetection to find the main contributors of the IMD3. When the optical carrier is modulated by a two-tone RF signal with angular frequency of ω_1, ω_2, and voltage of V_{RF} via a phase modulator (PM), the optical spectrum at the output of PM can be expressed as

$$E_{out}\left(t\right)=\sqrt{P_i}e^{j\omega_c t}\sum_{n=-\infty}^{+\infty}\sum_{k=-\infty}^{+\infty}J_n\left(m\right)J_k\left(m\right)e^{j\left(n\omega_1+k\omega_2\right)t} \tag{8}$$

where ω_c, P_i, J_n, $m = \pi \cdot V_{RF}/V_{\pi,RF}$, and $V_{\pi,RF}$ is the angular frequency of the optical carrier, input optical power, the n-th order Bessel function of the first kind, the modulation index of PM, and the RF half-wave voltage of the PM respectively. When the two-tone signal can be regarded as small signal, we can only take zero to second order sidebands into account to simplify the analysis.

The optical spectrum after spectral shaping can be written as

$$E_p\left(t\right)=\sqrt{P_i}e^{j\omega_c t} \cdot \left\{\begin{aligned}&\sqrt{A}\cdot J_0J_0\\&+\sqrt{A}\cdot J_{-1}J_1\left[e^{-j\left(\omega_1+\omega_2\right)t}+e^{j\left(\omega_1+\omega_2\right)t}\right]\\&+J_0J_1\left(e^{j\omega_1 t}+e^{j\omega_2 t}\right)\\&+J_{-1}J_2\left[e^{j\left(2\omega_1-\omega_2\right)t}+e^{j\left(2\omega_2-\omega_1\right)t}\right]\\&+\sqrt{A}\cdot J_{-1}J_0\left(e^{-j\omega_1 t}+e^{-j\omega_2 t}\right)\\&+\sqrt{A}\cdot J_{-2}J_1\left[e^{-j\left(2\omega_1-\omega_2\right)t}+e^{-j\left(2\omega_2-\omega_1\right)t}\right]\\&+J_0J_2\left(e^{j2\omega_1 t}+e^{j2\omega_2 t}\right)\\&+J_1J_1e^{j\left(\omega_1+\omega_2\right)t}\\&+\sqrt{A}\cdot J_{-2}J_0\left(e^{-j2\omega_1 t}+e^{-j2\omega_2 t}\right)\\&+\sqrt{A}\cdot J_{-1}J_{-1}e^{-j\left(\omega_1+\omega_2\right)t}\end{aligned}\right\} \tag{9}$$

where $J_n = J_n(m)(n = 0, \pm1, \pm2)$, $J_{-n} = (-1)^n J_n$. A is the power suppression to the optical carrier and the lower sideband.

The photocurrent of the RF signal detected from the processed optical spectrum can be expressed as

$$\begin{aligned}I_{PD}(t) &= R_{PD}|E_p(t)|^2\\&= I_1\cos\omega_{1,2}t + I_3\cos\left(2\omega_{1,2}-\omega_{2,1}\right)t\end{aligned} \tag{10}$$

where R_{PD} is responsivity of photodetector, I_1 and I_3 are the amplitude coefficients for fundamental signal and IMD3 components, which can be written as

$$I_1 \propto (\sqrt{A}-A)J_0^3J_1 + (1-\sqrt{A})J_0J_1^3 + (1-A)J_0^2J_1J_2 \tag{11}$$

$$I_3 \propto (A-\sqrt{A})J_0J_1^3 + (1-\sqrt{A})J_0^2J_1J_2 \tag{12}$$

when $m \ll 1$, $J_n(m) \approx m^n/(2^n n!)$, I_1 and I_3 can be expressed as

$$I_1 \propto (\sqrt{A}-A)m \tag{13}$$

$$I_3 \propto (2A-3\sqrt{A}+1)m^3 \tag{14}$$

To minimize the IMD3 distortion while maximizing the fundamental RF signal, the attenuation imposed to the optical carrier and lower sideband need to meet the requirements:

$$\begin{aligned} \sqrt{A} - A \neq 0 \\ 2A - 3\sqrt{A} + 1 = 0 \end{aligned} \tag{15}$$

The first requirement in Supplementary Equation (15) guarantees that the fundamental RF signal can be detected from photodetector. The second requirement describes the manipulation to the modulated spectrum for IMD3 terms cancellation. The second requirement in Supplementary Equation (15) has two solutions: A=1 and A=1/4. The solution A=1 means no suppression is imposed to the optical carrier and lower sidebad. In this case, neither fundamental signal nor IMD3 terms can be detected at the output of the photodetector. The other solution A=1/4 means the optical power of optical carrier and lower sideband should be attenuated for 6 dB, which is the linearization requirement we need. Under this condition, the IMD3 terms are greatly reduced while the fundamental signal can still be detected.

SUPPLEMENTARY INFORMATION F: OPERATIONAL PRINCIPLE OF SIMULTANEOUS FILTER AND LINEARIZATION

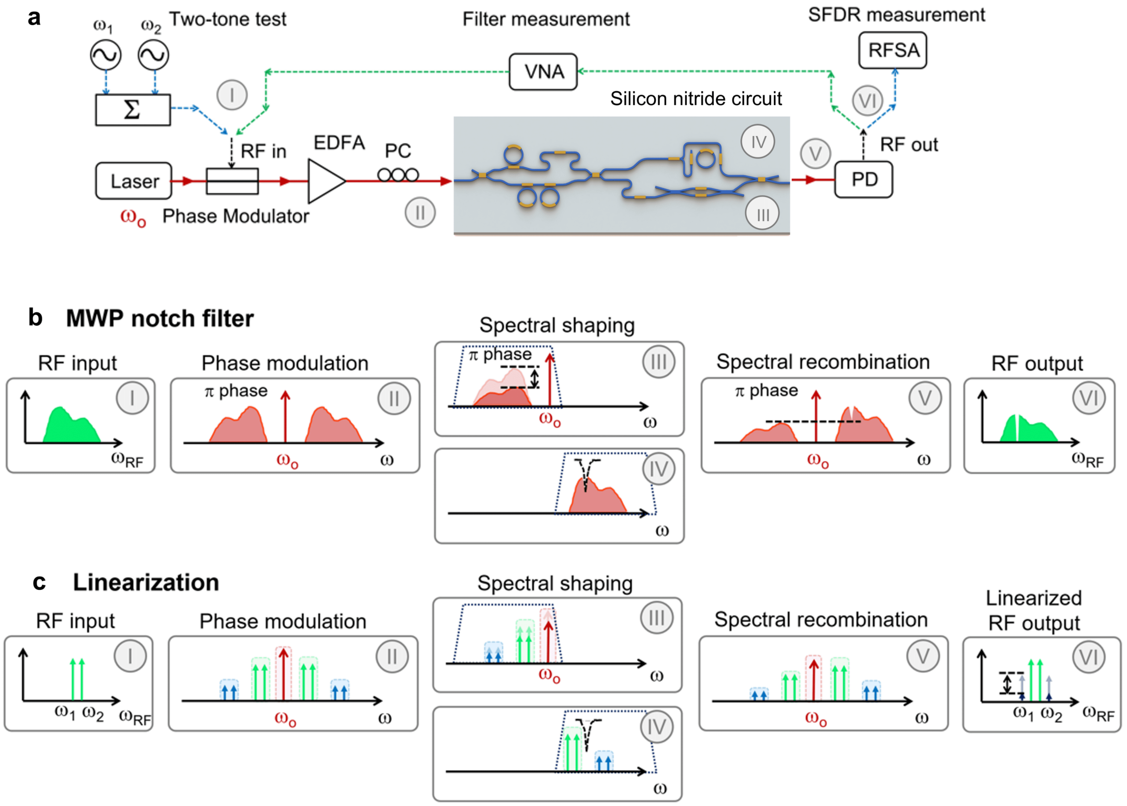

Supplementary Fig. 7. **Linearization of RF notch filter.** **(a)** Schematic of the experimental setup to demonstrate simultaneous notch filtering and linearization. A sweeping RF signal generated from a vector net work analyzer (VNA) is modulated to the optical carrier via a phase modulator (PM) and retrieved back to VNA from photodetector (PD) to demonstrate the notch filter functionality. A two-tone signal from two signal generators is combined and converted to optical domain via PM for two-tone test. The retrieved RF spectrum from PD is sent to RF signal analyzer (RFSA) for third-order intermodulation distortion (IMD3) and spurious-free dynamic range (SFDR) measurement. **(b)** Signal flow for notch filter formation. The filter is formed through RF cancellation at the notch frequency. **(c)** Signal flow for the simultaneous linearization. The amplitude and phase of the optical carrier and first and second order sidebands are adjusted to achieve cancellation of IMD3 products at the output. EDFA: erbium-doped fiber amplifier, PC: polarization controller.

In this experiment, we experimentally demonstrated, for the first time, simultaneous RF notch filter and linearization using a combination of the MT and the DI-RR in a single photonic chip. The DI-RR is set to create a notch response, while The MT works for two purposes, synthesize desired modulations spectrum needed for RF notch filter and linearization. We conducted the experiment with setup as depicted in Supplementary Fig. 7(a) with the operating principle of simultaneous RF notch filter and linearization as illustrated in Supplementary Fig. 7(b) and 7(c).

The RF notch filter here is synthesized with the basis of phase cancellation [7]. First, the RF signal (I) is phase modulated into the optical carrier generating single frequency optical carrier with multiple sidebands that are out-of-phase (II). The phase modulation spectrum then coupled into the programmable integrated MWP circuit containing of the MT and DI-RR. The spectral de-interleaver in the MT spatially separates the phase modulation spectrum into two parts, where the optical carrier and lower sideband are attenuated by the tunable attenuator (III). The upper sideband is processed by notch response from the DI-RR set at under-coupling (UC) regime to equalize the amplitudes of two sidebands at designate frequency (IV). Next, the optical spectrum are recombined at the output of the chip, creating an asymmetric dual sideband (aDSB) modulation with anti-phase relation (V). After photodetection, a high rejection RF notch filter is achieved, as the mixing products of the sidebands and the optical carrier are destructively interfered at the notch frequency (VI).

When the high rejection notch filter is achieved, we implement a two-tone test at the passband of the RF notch filter for the linearization process. The two-tone RF signal (I) is phase modulated into the optical carrier, generating multi-order sidebands at both sides of the optical carrier (II). Under the small-signal condition, we take ±1 and ±2 order sidebands into account for approximation. Then, the optical spectrum is coupled to the photonic chip, where the spectral de-interleaver split the optical modulation spectrum into two channels. One channel contains the optical carrier, the -1 order and -2 order sidebands, which are manipulated by a cascaded tunable attenuator and a phase shifter. The attenuator suppresses the power of the optical signal in this channel to meet the linearization condition in Supplementary Equation (15). The phase shifter adjusts the relative phase relationship between two channels to ensure the phase relation of the optical spectrum at the output of the chip is still the phase modulation scheme (III). The other channel consists of $+1$ and $+2$ order sidebands (IV). The $+1$ order sideband of the two-tone signal is in the passband of the RF filter and the $+2$ order sideband of the two-tone is out of the working bandwidth of the RF filter. The DI-RR used to tailor the frequency components at the stopband will not have effects on the two-tone signal. Then, the manipulated spectrum is recombined at the output of the chip, generating an aDSB modulation that satisfies the linearization condition and RF notch filter requirement simultaneously (V). This recombined spectrum is sent to the photodetector, resulting in an RF spectrum with suppressed IMD3 terms due to the destructive interference between the mixing products of the multi-order sidebands and the optical carrier (VI).

SUPPLEMENTARY INFORMATION G: EXTENDED MEASUREMENTS OF IMD3 SUPPRESSION AND SFDR

To further characterize the performance of our proposed linearized RF notch filter, we extended our measurements of IMD3 suppression and SFDR when the two-tone test frequency and the notch filter frequency was tuned separately. We first fixed the notch frequency at 12 GHz and performed two-tone measurements at 8 GHz, 9 GHz, 10 GHz, and 16 GHz. The results of the IMD3 suppression are shown in Supplementary Fig. 8. It is clear that the IMD3 terms are greatly suppressed for more than 28 dB in all of these two-tone frequencies.

The results of the SFDR at different frequencies are shown in Supplementary Fig. 9. In all the cases, SFDR of more than 122 $\mathrm{dB} \cdot \mathrm{Hz}^{2/3}$ are observed with improvements around 20 dB compared with nonlinearized states.

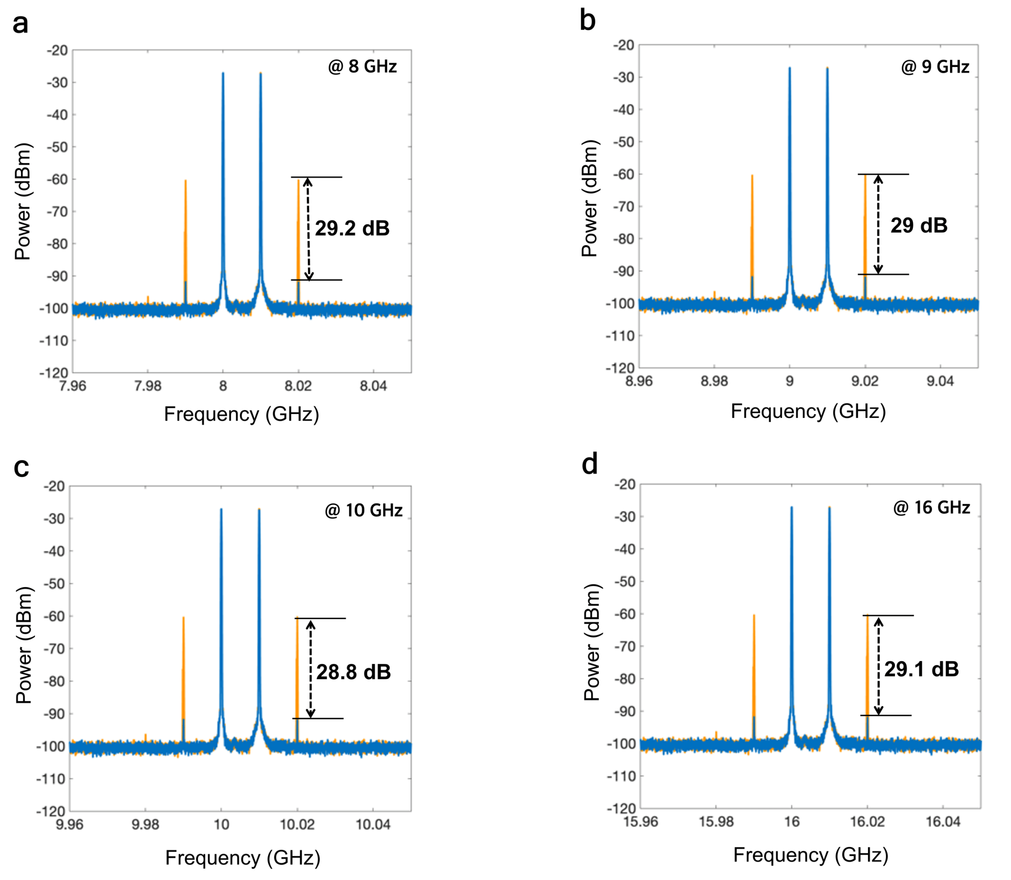

Supplementary Fig. 8. **Third-order intermodulation distortion (IMD3) suppression at various two-tone frequencies with notch response at 12 GHz.** **(a)** two-tone signal at 8 GHz **(b)** two-tone signal at 9 GHz **(c)** two-tone signal at 10 GHz **(d)** two-tone signal at 16 GHz.

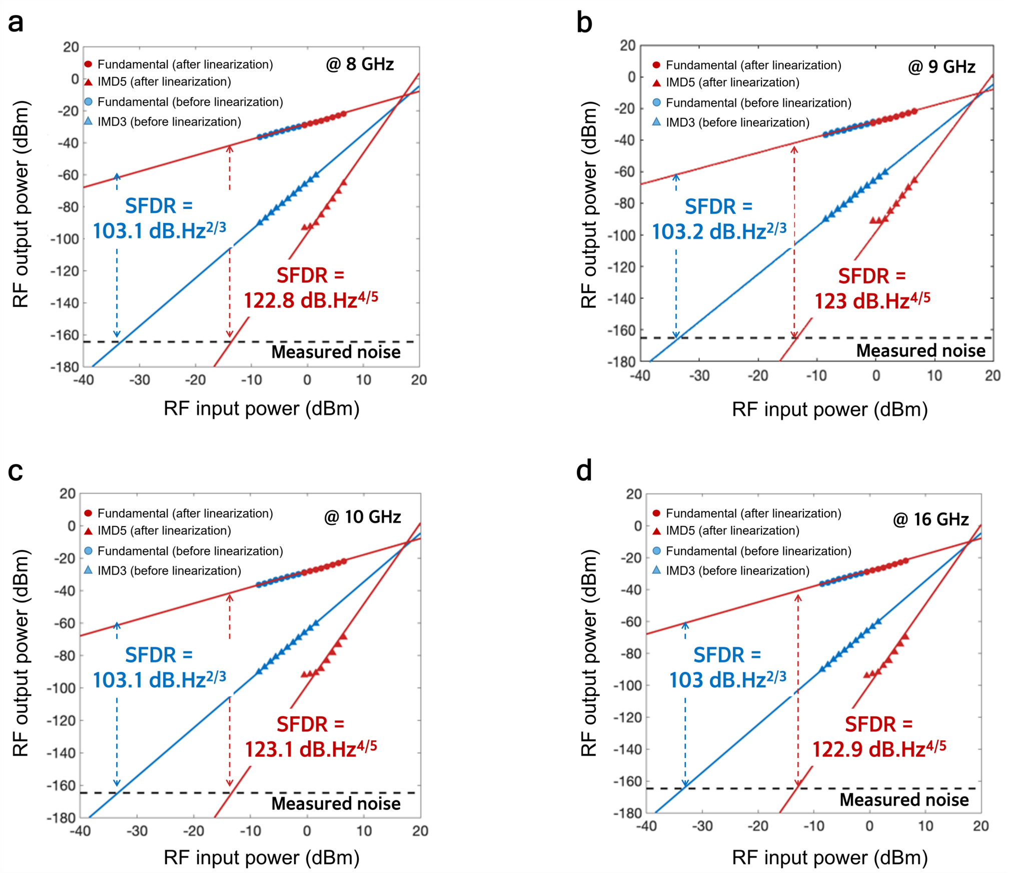

Supplementary Fig. 9. **Spurious-free dynamic range (SFDR) measurements at various two-tone frequencies with notch response at 12 GHz.** **(a)** SFDR at 8 GHz **(b)** SFDR at 9 GHz **(c)** SFDR at 10 GHz **(d)** SFDR at 16 GHz. IMD3: third-order intermodulation distortion, IMD5: fifth-order intermodulation distortion.

[1] Luo, L.-W. *et al.* High bandwidth on-chip silicon photonic interleaver. *Optics express* **18**, 23079–23087 (2010).
[2] Guo, X. *et al.* Versatile silicon microwave photonic spectral shaper. *APL Photonics* **6**, 036106 (2021).
[3] Daulay, O., Liu, G., Guo, X., Eijkel, M. & Marpaung, D. A tutorial on integrated microwave photonic spectral shaping. *Journal of Lightwave Technology* **39**, 700–711 (2021).
[4] Bogaerts, W. *et al.* Silicon microring resonators. *Laser & Photonics Reviews* **6**, 47–73 (2012).
[5] Zhuang, L. *et al.* Low-loss, high-index-contrast si 3 n 4/sio 2 optical waveguides for optical delay lines in microwave photonics signal processing. *Optics express* **19**, 23162–23170 (2011).
[6] Cohen, R., Amrani, O. & Ruschin, S. Response shaping with a silicon ring resonator via double injection. *Nature Photonics* **12**, 706–712 (2018).
[7] Marpaung, D. *et al.* Si 3 n 4 ring resonator-based microwave photonic notch filter with an ultrahigh peak rejection. *Optics express* **21**, 23286–23294 (2013).
